# Leveraging smallholder livestock production to reduce anemia: A qualitative study of three agroecological zones in Ghana

**DOI:** 10.1016/j.socscimed.2018.07.028

**Published:** 2018-09

**Authors:** Hanson Nyantakyi-Frimpong, Esi K. Colecraft, Raphael Baffour Awuah, Leonard Kofi Adjorlolo, Mark L. Wilson, Andrew D. Jones

**Affiliations:** a Department of Geography & the Environment, University of Denver, Denver, CO 80210, USA; b Department of Nutrition and Food Science, University of Ghana, Legon-Accra, Ghana; c Regional Institute of Population Studies, University of Ghana, Legon-Accra, Ghana; d Livestock and Poultry Research Centre, School of Agriculture, University of Ghana, Legon-Accra, Ghana; e Department of Epidemiology, School of Public Health, University of Michigan, Ann Arbor, MI 48109, USA; f Department of Nutritional Sciences, School of Public Health, University of Michigan, Ann Arbor, MI 48109, USA

**Keywords:** Animal-source foods, Anemia, Nutrition, Livestock production and management, Infectious diseases, Ghana

## Abstract

Livestock production and Animal-Source Foods (ASFs) like meat, milk, and eggs are excellent sources of essential micronutrients, including iron and zinc. There is evidence that encouraging increased access to and consumption of these ASFs may either positively or negatively impact anemia, or have no nutritional effects. Drawing upon first-hand experiences in Ghana, this study sought to: (1) identify the main motivations for raising livestock in Ghana; (2) describe the major barriers to consuming ASFs, especially among women of reproductive age (WRA); and (3) explore the feasibility of different livestock-centered interventions to reduce anemia. Key informant interviews and focus group discussions were held with relevant stakeholders at different geographical scales - the national, regional, district, and community levels. The results suggest that livestock enable savings, allow resource-poor households to accumulate assets, and help finance planned and unplanned expenditures (e.g., school fees and illness). Due to these multiple and often pressing uses, direct consumption of home-reared ASFs is not a major priority, especially for poor households. Even when ASFs are consumed, intra-household allocation does not favor women and adolescent girls, demographic groups with particularly high micronutrient requirements. The study participants discussed possible interventions to address these challenges, including (1) increasing livestock ownership through in-kind credit; (2) encouraging nutrition-related behavior change; (3) improving livestock housing; and (4) hatchery management. The paper discusses these interventions based upon potential acceptance, feasibility, cost effectiveness, and sustainability in the Ghanaian context.

## Highlights

• Animal-Source Foods (ASFs) are excellent sources of micronutrients.
• This study identifies the main motivations for raising livestock in Ghana.
• The report also describes barriers to consuming ASFs, especially among women.
• Results suggest diverse, non-nutritional reasons for raising livestock.
• The analysis highlights possible interventions to increase ASF consumption.

## 1 Introduction

Anemia is among the most common global nutritional disorders and public health concerns. Two billion people worldwide are anemic, and disproportionately so in women of reproductive age (WRA) and young children (World Health Organization, 2015). Global anemia prevalence is 38% in pregnant women (32.4 million) and 29% in non-pregnant women (496 million) (Stevens et al., 2013), with marked variation among regions. Indeed, nowhere is anemia prevalence higher than in sub-Saharan Africa (SSA). The World Health Organization estimates that 38% and 46% of non-pregnant and pregnant WRA, respectively, are anemic in SSA (World Health Organization, 2015). Especially for WRA, anemia is of grave concern since it contributes to adverse birth outcomes, including low birth weight, stillbirth, preterm birth, and increased child and maternal mortality (Galloway et al., 2002; Stevens et al., 2013).

Many factors underlie anemia risk, including parasitic infections, inherited blood disorders, numerous vitamin deficiencies (e.g., folic acid, vitamins B12 and A) (Balarajan et al., 2011), and acute or chronic immune activation (Weiss and Goodnough, 2005). However, iron deficiency is the most common cause of anemia (World Health Organization, 2015). Iron-deficiency anemia interventions have centered primarily on nutrition-specific approaches (e.g., dietary or micronutrient supplementation (Galloway et al., 2002; Vieira et al., 2017)), targeting the most proximate determinants of the problem (Bhutta et al., 2013). Much attention has also been given to fortification of cereals and other food crops, for example, production of iron-rich beans and pearl millet (Haas et al., 2013). Despite evidence of benefits, several factors have hindered the effectiveness of these interventions including supply chain challenges, and the difficulty of reaching target populations (Bhutta et al., 2013).

Nutrition-sensitive interventions, in contrast, focus on the underlying determinants of nutrition, including poverty, household food insecurity, and unhealthy living environments (Ruel et al., 2013). Such interventions have in part included the promotion of increased consumption of fruits and vegetables through homestead gardens (Helen Keller International, 1993). There is evidence, however, that livestock-based interventions may effectively complement such interventions focused on plant-source foods by promoting consumption of animal-source foods (ASFs) (Berti and Cossio, 2017; Leroy and Frongillo, 2007; Nicholson et al., 2003). Case studies increasingly suggest that greater consumption of ASFs may sustainably diversify and improve diet quality, nutritional status, and overall health (Osei et al., 2016; Rawlins et al., 2014). Iron intake could be enhanced by increasing the availability and consumption of home-produced ASF (Murphy and Allen, 2003). ASFs such as beef, fish, and poultry are rich sources of bioavailable heme iron, which is more easily absorbed than the iron contained in plants (Murphy and Allen, 2003; Zhang et al., 2016). Also, the selling of self-produced animals or animal products can increase household income that then could be used to purchase micronutrient-rich foods (Nicholson et al., 2003).

**Fig. 1:**
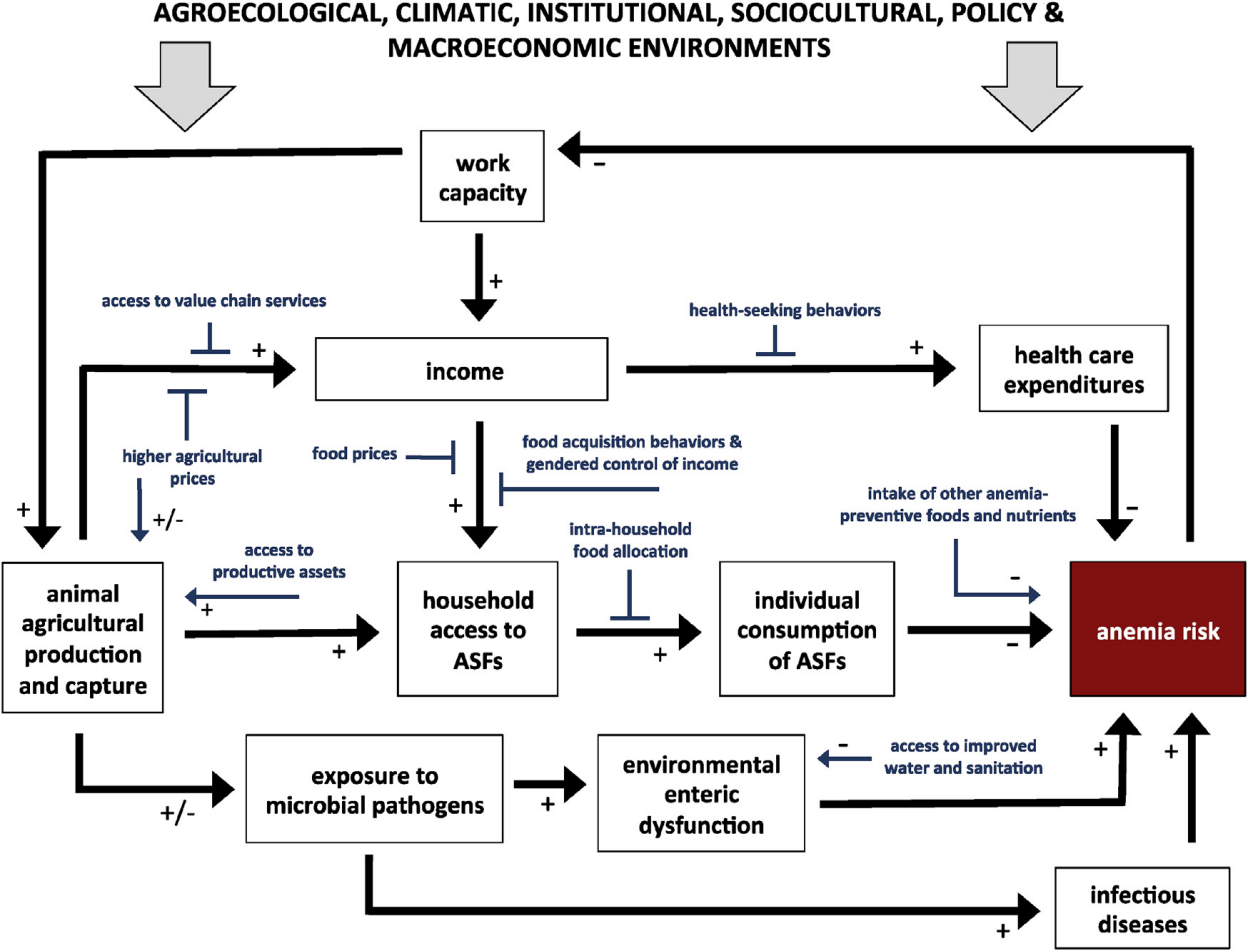
Conceptual framework of hypothesized causal linkages between animal agricultural production and capture, and anemia among adolescent girls and women of reproductive age.

Despite the nutritional benefits of ASFs, livestock production may also negatively influence anemia risk (Fig. 1). First, not all ASFs are rich in iron. Cow milk, for example, contains little iron, and can actually enhance iron-deficiency among infants and toddlers through intestinal bleeding and competition of calcium with iron (Ziegler, 2011). Second, because women and girls commonly bear animal husbandry responsibilities, increased livestock production may decrease the amount and quality of time available for child care and feeding activities (Njuki et al., 2016). Third, keeping livestock could also increase risk of clinical and sub-clinical infection, either through contact with feces, or contamination of food and water sources (Randolph et al., 2007). The close proximity of domestic livestock to humans has been associated with transmission of *Campylobacter*, causing acute bloody diarrhea (Harvey et al., 2003). This is particularly problematic for young children, among whom fecal-oral transmission may commonly occur during play (Marquis et al., 1990). In sum, the multiple causal pathways involved make it difficult to accurately determine how livestock production may affect anemia risk in a given setting.

Accordingly, this study aimed to better understand the prospects and challenges of raising livestock to reduce anemia, while not increasing exposure to adverse health threats. Drawing upon a subset of qualitative data collected from experienced Ghanaian stakeholders, we sought to:

1. Identify the main motivations for raising livestock in Ghana;
2. Describe the major barriers to consuming ASFs, especially among WRA; and
3. Explore the feasibility of different livestock-centered interventions to reduce anemia through diverse pathways.

The conceptual framework in Fig. 1 is motivated by an evidence-based understanding of the linkages between animal agricultural production and capture, and anemia (e.g., Leroy and Frongillo, 2007; Nicholson et al., 2003). This case study was therefore designed to understand whether and how any of the casual linkages in the conceptual framework plays out across different regions of Ghana. As an open-ended qualitative study, however, we did not restrict data collection and analyses to only the casual linkages highlighted in the framework (e.g., food prices, food acquisition behaviors, gendered control over food, exposure to microbial pathogens, and infectious diseases. Although these casual linkages informed the design of data collection instruments and analysis, study participants were also allowed to talk about what is of greater concern to them. Indeed, the framework neither shows all moderating factors along the different pathways, nor is it meant to serve as a program theory framework for a proposed future intervention. Rather, it illustrates the broad impact pathways that we hypothesize will be relevant to understanding the potential for animal production and capture to affect anemia among WRA. The framework also demonstrates the complex network of factors that need to be considered in designing interventions aimed at reducing anemia among WRA.

## 2 Methods

### 2.1 Geographic setting

**Fig. 2:**
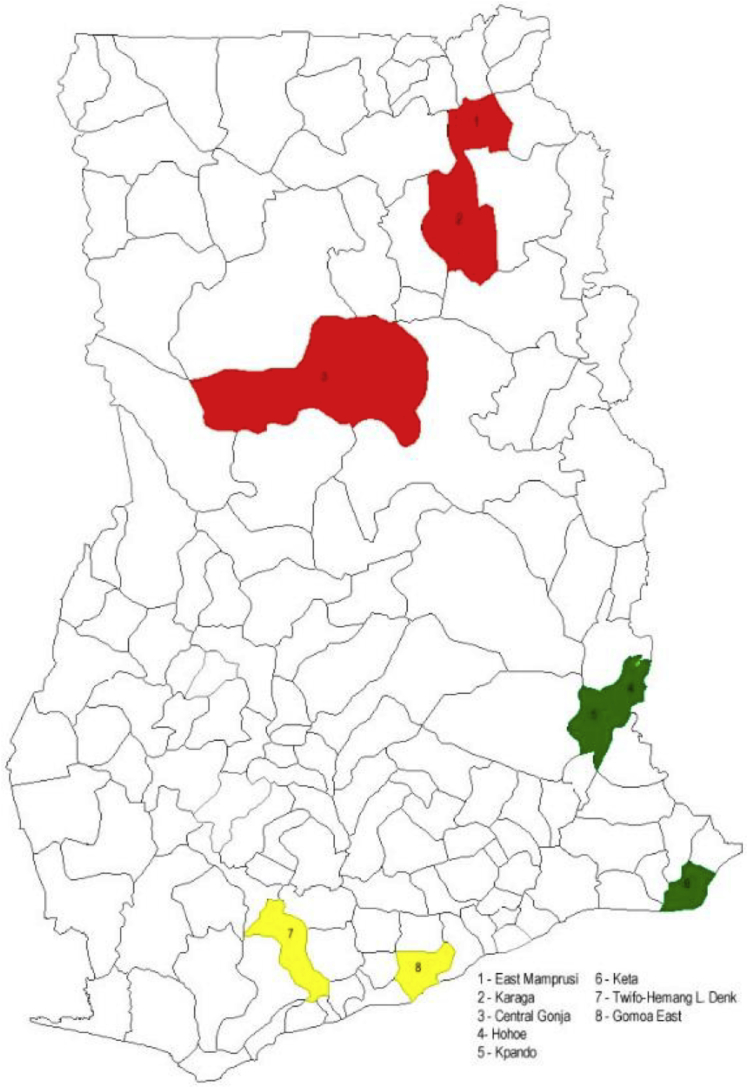
Locations of the study Districts and Regions in Ghana. ***Note*:** Districts in red, green, and yellow are located in the Northern, Volta, and Central Regions, respectively. (For interpretation of the references to colour in this figure legend, the reader is referred to the Web version of this article.)

This study was conducted in Ghana, which has among the highest anemia prevalence in the world (Stevens et al., 2013) - 42% of Ghanaian women aged 15–49 years, and 66% of children aged 6–59 months are anemic (Ghana Statistical Service, 2015a, Ghana Statistical Service (GSS) et al., 2015b). Furthermore, Ghana's distinct ecological environments and diverse animal husbandry practices make it an ideal context for exploring the links among livestock production, ASF consumption, infection risks, and anemia. Field research was undertaken in three Regions of the country that represent this diversity (i.e., Central, Northern and Volta) and that also have the country's highest anemia prevalence (Fig. 2; Table 1).

**Table 1:** Characteristics of the three study Regions in Ghana.

Characteristics	Region		
	*Central*	*Volta*	*Northern*
Total population (2010 national census)	2,201,863^a^	2,118,252^b^	2,479,461^c^
Mean household size, number of persons	4^a^	4.2^b^	7.7^c^
Population living below the poverty line ($1.83 per day), %^d^	18.8	33.8	50.4
Agricultural households engaged in livestock rearing, %	34.9^a^	3.9^b^	30.0^c^
Agricultural households engaged in fish farming, %	0.2^a^	0.2^b^	0.1^c^
Anemia (chiuldren under age 5), %^e^	85.4	79.5	82.0
Study Districts	1. Gomoa East 2. Twifo-Hemang-Lower Denkyira	1. Hohoe 2. Keta 3. Kpando	1. Central Gonja 2. East Mamprusi 3. Karaga
Study communities	*n* = 4	*n* = 6	*n* = 6
Major ecological zone	Coastal savannah	Savannah-forest transition	Guinea savannah
Number of growing seasons	2 seasons; a major and minor rainy season	2 seasons; a major and minor rainy season	1 season; a single and relatively short rainy season
Major livelihood activities	Farming, fishing, hunting, livestock rearing	Farming, fishing, hunting, livestock rearing	Farming, livestock rearing

Data Sources.

a Ghana Statistical Service (2013a, p.22, 38, 112).

b Ghana Statistical Service (2013c, p.24, 115, 124).

c Ghana Statistical Service (2013b, p.24, 38, 91).

d Ghana Statistical Service (2014, p.14, p.14).

e Ewusie et al. (2014, p.7); Children under age 5 with hemoglobin level less than 11.0 g/dl were considered anemic in this cited study. The cut-off values for the various levels of severity were: <7.0 g/dl for severe anemia, 7.0 g/dl-9.9 g/dl for moderate anemia, and 10.0 g/dl-10.9 g/dl for mild anemia.

### 2.2 Study design and ethical review

For this formative study, we used an interpretative methodology that encourages the critical involvement of community members in problem appraisal, analysis, and intervention planning (Farquhar et al., 2006; Miles et al., 2014). The study protocol was reviewed and approved by the Institutional Review Boards at the University of Michigan (Protocol # HUM00120734), University of Ghana (Protocol # ECBAS 001/16–17) and Ghana Health Service (Protocol # GHS-ERC 07/01/17). Participation was voluntary and required informed written consent. Fieldwork was conducted by trained moderators and field assistants with expertise in conducting qualitative interviews in English and local Ghanaian languages.

### 2.3 Data collection

Data were collected in two phases, and came from key informant interviews (KIIs) and focus group discussions (FGDS). In Phase One (November–December 2016), we conduced KIIs with relevant national-level stakeholders in Accra, Ghana. Purposive maximum variation sampling was used to identify participants. This sampling involved the strategic recruitment of information-rich participants with varying experiences (Patton, 2014). Using this approach, we recruited 13 key informants from government and Non-Governmental Organizations (NGOs) working in the areas of agriculture (*n* = 6) or health and nutrition (*n* = 7) (Table 2). Interview questions were developed using two separate guides, one each for agriculture- and health-related stakeholders. The questions were developed around a conceptual model (Fig. 1), with a specific focus on anemia risks among women and adolescent girls. Interviews lasted 50–60 min, were audio recorded with permission, and then transcribed for analysis. The research team discussed initial findings from the analysis, and then planned the second phase of fieldwork.

**Table 2:** Total number of Key Informant Interviews (KIIs) and Focus Group Discussions (FGDs).

Setting in Ghana	Key Informant Interviews (KIIs)	Focus Group Discussions (FGDs)
National	13	–
Central Region	9	16
Northern Region	10	16
Volta Region	14	16
***Total***	***46***	***48***

Note: Not this entire dataset is used in the current paper. We draw on only a sub-sample (see Table 3).

Phase Two (February–March 2017) involved KIIs and FGDs conducted at the regional, district, and community levels. One of the main purposes of this phase was to triangulate qualitative data (Patton, 2014), as well as to complement and deepen information gathered at the national level. A multi-stage site sampling approach was used, involving the selection of districts and communities within each of the three study regions. This sampling approach was implemented in close consultation with local experts from the Ghana Health Service, and the Ministry of Food and Agriculture. First, we purposively selected districts that are representative, but also show some variation in anemia prevalence and livestock/fishing activities. Using this sampling approach, eight Districts were selected, including three in Northern Region, three in Volta Region, and two in Central Region (Table 1). Then, within each of the eight District, two communities were purposively selected to represent different characteristics using the following criteria: (1) availability of ASFs that are typical in that Region; (2) presence of livestock-rearing activities; and (3) physical accessibility to the community. We gained access to these 16 communities through District officials from the Ghana Health Service and the Ministry of Food and Agriculture.

During Phase Two, 33 KIIs were conducted with relevant regional and district stakeholders (*n* = 9 in Central Region; *n* = 10 in Northern Region; and *n* = 14 in Volta Region). As well, 48 FGDs (*n* = 16 in each region) were held to assess areas of consensus and divergence among community members (Table 2). Using principles of maximum variation, we recruited FGD participants based on gender and age. Separate FGDs were held with men (aged ≥20 years), adolescent girls (aged 15–19 years), and adult women (aged 20–49 years) in each of the 16 communities. This disaggregation was crucial, particularly in a context where women and adolescent girls tend to feel uneasy when discussing gender-sensitive issues in the presence of elderly men. To ensure consistency across the FGDs, we developed a focus-group guide based upon our research objectives, and emerging themes from the first phase of the fieldwork. Focus groups included 7–11 participants, lasted ∼1 h, were audio recorded with permission, and were later transcribed for analysis.

### 2.4 Data analysis

Qualitative data analysis followed the methods outlined by Miles et al. (2014) and Patton (2014). Hand-coding was used to ensure deep and continued immersion in the qualitative data. Firstly, a coding scheme was developed using our conceptual model. Secondly, codes or labels were assigned to segments of transcripts to help catalogue key concepts while preserving the context in which these concepts occur. After reaching theoretical saturation in the coding process (i.e., when no new concepts emerged from successive coding), we developed emerging themes. Themes represented patterned responses or meanings within the dataset (Miles et al., 2014). Key themes were identified according to criteria that included: (1) relevance to the research objectives; (2) frequency that the theme was mentioned; and (3) the predominance of the same theme across different types of participants (Miles et al., 2014; Patton, 2014). An interdisciplinary team of five people was directly involved in the data analysis. Each person performed an independent coding using the coding scheme. Team discussions were then held daily to confer on how individual or disciplinary biases may have affected the codes generated. All inter-coder inconsistencies were resolved during these daily meetings. Midway through the analysis, a conference call was also organized among the entire study team in Ghana and the United States to further discuss and reach consensus on inter-coder inconsistencies. To ensure external validity of the coded themes, we organized a feedback workshop in Accra, Ghana, on 13 June 2017 to share preliminary results with stakeholders. Thirty-five people attended this ‘member-checking’ workshop (Miles et al., 2014), 13 of whom had taken part in our KIIs, and 14 in our FGDs. The remaining 8 participants were scholars conducting similar research at the University of Ghana, University of Development Studies, and University of Cape Coast. The datasets were not lumped together at the member-checking workshop. Rather, we gave four different presentations at the workshop, two of which focused specifically on findings from the KIIs and FGDs. The findings from the KIIs and FGDs were fully validated by their respective participants.

For this article, analysis was undertaken on 22 FGDs and 21 KIIs, which is a subset of the full dataset from the broader study (46 KIIs and 48 FGDs [Table 2]). In arriving at this sub-sample, several factors were taken into consideration, including conceptual and methodological best practices in qualitative research (Fusch and Ness, 2015; Miles et al., 2014; Patton, 2014; Sandelowski, 2008). We excluded transcripts that (1) did not contain any information relevant to the main objectives of this current article; or (2) comprised redundant responses that did not provide information-rich transcripts to inform analyses (Sandelowski, 2008). We also assessed the adequacy of the sub-sample by considering inductive thematic saturation, that is, the point when no new codes or themes emerge from adding new transcripts to the analyses (Patton, 2014; Saunders et al., 2017). It is important to emphasize that the chosen sub-sample sufficiently represents each of the stakeholder groups interviewed, including respondents from the national, regional, and community levels. Gender and age differences are also adequately represented. Moreover, from a qualitative research standpoint, a sub-sample that includes an experienced group of 21 key informants, as well as 203 diverse FGD participants, is considered adequate for exploratory studies (e.g., see Fusch and Ness, 2015; Miles et al., 2014; Patton, 2014).

## 3 Results

### 3.1 Sample population characteristics

Selected characteristics of the study population showed broad variations across age, gender, region of residence, and stakeholder expertise (Table 3). Of the community focus group participants, mean age was 18 years (adolescent girls), 37 years (other WRA), and 42 years (men). Key informants from the national, regional and district levels had 5–35 years of work experience in the fields of agriculture and/or health.

**Table 3:** Sample characteristics (*N* = 224 participants; 21 in KIIs, and 203 in FGDs).

KII#	Profile	Age	Gender	Setting
1	Veterinary Officer	–	Female	National Level (*n* = 7 participants)
2	Gender Specialist	–	Female	
3	Nutritionist	–	Female	
4	NGO Officer	–	Male	
5	Animal Production Specialist	–	Male	
6	Agricultural Extension Specialist	–	Male	
7	Health NGO Officer	–	Male	
8	Health NGO Officer	–	Female	Central Region (*n* = 5 participants)
9	Chairman, Community-based Organization#2	–	Male	
10	Chairman, Community-based Organization#1	–	Male	
11	Medical Officer	–	Male	
12	NGO Officer	–	Male	
13	Medical Officer	–	Male	Northern Region (*n* = 4 participants)
14	Food and Agriculture Development Specialist	–	Male	
15	NGO Officer	–	Male	
16	Organizing Officer, Local Farmers' Group	–	Male	
17	Fisheries Specialist	–	Female	Volta Region (*n* = 5 participants)
18	Chairman, Community-based Organization	–	Male	
19	Medical Officer	–	Male	
20	Food and Agriculture Development Specialist	–	Male	
21	NGO Officer	–	Male	

**FGD#**	**Profile of Participants**^a^	**Mean Age (Years)**	**Gender**	**Setting**
1	Community Members (*Adolescents*) (*n* = 9)	18	Female	Central Region (*n* = 6 FGDs; 3F, 3M)
2	Community Members (*Adolescents*) (*n* = 10)	17	Female	
3	Community Members (*n* = 8)	42	Female	
4	Community Members (*n* = 9)	46	Male	
5	Community Members (*n* = 10)	38	Male	
6	Community Members (*n* = 10)	42	Male	
7	Community Members (*Adolescents*) (*n* = 9)	18	Female	Northern Region (*n* = 9 FGDs; 6F, 3M)
8	Community Members (*Adolescents*) (*n* = 10)	19	Female	
9	Community Members (*Adolescents*) (*n* = 10)	17	Female	
10	Community Members (n = 9)	33	Female	
11	Community Members (*n* = 9)	42	Female	
12	Community Members (*n* = 10)	33	Female	
13	Community Members (*n* = 9)	39	Male	
14	Community Members (*n* = 8)	42	Male	
15	Community Members (n = 9)	48	Male	
16	Community Members (*Adolescents*) (*n* = 10)	17	Female	Volta Region (*n* = 7 FGDs; 4F, 3M)
17	Community Members (*Adolescents*) (*n* = 11)	18	Female	
18	Community Members (*n* = 11)	37	Female	
19	Community Members (*n* = 9)	33	Female	
20	Community Members (*n* = 8)	39	Male	
21	Community Members (*n* = 8)	41	Male	
22	Community Members (*n* = 7)	47	Male	

F = Female; M = Male.

a Note: *n* = number of participants in the focus group. A total of 203 participants in the 22 groups combined.

### 3.2 Overarching themes of responses

The study findings are organized around three overarching themes (Table 4), corresponding with the three specific study objectives. Firstly, we describe the different motivations for raising livestock (Objective #1). Secondly, we present data on the major barriers to consuming ASFs, especially among WRA (Objective #2). Finally, based upon stakeholders' experience and field evidence, we present data on identified livestock interventions that could help reduce anemia in Ghana (Objective #3).

**Table 4:** Summary of overarching themes.

Organizing themes	Basic themes	Number of KIIs or FGDs in which theme was mentioned	
		KIIs (*n* = 21)	FGDs (*n* = 22)
**Theme 1**. Multiple, diverse non-nutritional reasons for raising livestock	i. Income generation & savings (school fees, health, emergency funds)	19	22
	ii. Ceremonial or symbolic uses (marriages, funerals, child naming)	18	19
	iii. Enhancing crop production (draught animal power; manure fertilizer)	16	13
**Theme 2**. ASF affordability barriers and poor understanding of nutritional values	i. Difficult to afford (production risks and costs, price at market high)	16	11
	ii. Lack of knowledge (special nutritional needs, ASF health benefits)	11	6
	iii. Selective consumption (mostly small or sick animals, unequal sharing at the household level [gender and age])	13	18
**Theme 3**. Perceived pathways for expanding ASF consumption to reduce anemia	i. Social and behavior change communication (education)	17	5
	ii. Improved livestock housing (animal safety, reducing human disease)	10	11
	iii. Encourage livestock with in-kind credit (expand programs, education)	7	10
	iv. Enhanced fowl hatchery management (share improved techniques)	2	–

#### 3.2.1 Theme 1: Multiple, diverse, non-nutritional reasons for raising livestock

The use of livestock and their products as a source of income was the most prevalent response in the study. Respondents frequently talked about livestock in commercial terms, seeing it as a bank account or source of emergency funds, rather than for consumption and nutrition:

The households look at the livestock as a bank. Because they wait until there's some emergency or someone has died and they need it for the funeral. But for it to be an everyday thing to slaughter a cow and consume the meat, no **[“Damien,”**^1^**NGO Key Informant, National Level]**.

From the interviews, it was very clear that income generated from livestock sales was being used to finance education, healthcare, and farm labor:

We sell the livestock to take care of our children in school, paying their school *fees*. We also sell them to *support* our farming activities, paying for labor on the farm. If we are sick and we need money for the hospital, we also sell the livestock **[“Leo,” 30 years, Men's FGD#13, Northern Region]**.

Human consumption of livestock was also mentioned in the interviews, though this was restricted mainly to eggs, chickens, and guinea fowls. Even among households consuming poultry, this was reported to occur infrequently, and restricted to special social occasions:

We slaughter chicken and guinea fowls occasionally at home for consumption. But we will not slaughter a goat or a sheep just for home consumption. We also produce some of the eggs at home. We mostly purchase meat and milk for home consumption **[“Susan,” 45 years, Women's FGD#19, Volta Region]**.

The desire for income was tightly interwoven into narratives explaining these consumption patterns. Moreover, home consumption of sheep and goat was said to be rare, and the consumption of home-grown cattle was never mentioned in any interview. Consumption of small and large ruminants typically occurred if the animal became sick or died. Repeatedly, participants said that livestock-rearing households would not normally consume a healthy animal, often citing the need for income as the main reason:

The farmers want money, so they would not want to consume the livestock. Today, everything is about money. No one would want to slaughter a healthy livestock for consumption **[“Palmer,” CBO Key Informant, Central Region]**.

Non-income benefits were also mentioned in the interviews and focus groups. For some households, livestock are used for ploughing, and manure production for soil fertility management. For others, livestock have ceremonial or symbolic uses, including marriage contracts (e.g. bride wealth). Livestock manure also reportedly had other uses, including serving as a mosquito repellent from cow dung that is mixed with water and smeared around homesteads.

#### 3.2.2 Theme 2: Major barriers shaping access to and consumption of ASFs

Participants also discussed major barriers and disincentives that limit ASF consumption. One prominent concern was ignorance about the nutritional value of ASFs. Some respondents revealed that ASF consumption may be low because there is a lack information on the nutritional benefits of these foods:

As I said earlier, there may be certain animal products that are good for people like pregnant women. But most people don't know these nutritional benefits. That's why they don't consume a lot of meat. If I'm consuming goat meat and chicken, I'm not thinking about the nutrients from these foods. My goal is to just eat and be full. […] As farmers, we don't think about meat in that way [in terms of nutritional benefits] **[“Quincey,” 33 years, Men's FGD#6, Central Region]**.

For non-livestock-rearing households in particular, affordability was another significant consideration whether or not ASFs were consumed. Many participants felt that ASFs are extremely expensive, particularly for poor households. Similarly, others remarked that availability of ASFs is not a key challenge, as meat, milk, and eggs are abundant in markets. However, their costs are highly prohibitive:

On a scale right now, people are not drinking milk, eating eggs or eating enough meat. Not because they are not on the market. They are. But we can't buy. We can't afford. The price is high […]. If things are fine and a boiled egg is 20 pesewas [∼US$ 0.04] or 30 pesewas [∼US$ 0.07], you will see the number of people eating eggs like nobody's business. That's the fact **[“Morgan,” Government Key Informant, National Level]**.

Some ASFs were considered to be so expensive that their consumption is seen as a luxury. This was particularly raised in reference to Guinea fowl, snails, beef, and mutton. Study participants recognized that the high prices of ASFs are tied to costs of production, which are then passed onto consumers:

Now let's take meat - to feed the livestock and the drugs for their management is very expensive. What this means is that a kilogram of beef or mutton on the market is very high. People cannot afford it. So, you go to the market or the butchery. The meat is there. The butchers have slaughtered the animals, but the customers are not buying. The customer can't buy one kilogram of mutton or beef. Cost of production is so high. No wonder we don't eat enough meat **[“Mindy,” Government Key Informant, National Level]**.

In the limited situations where livestock was consumed, participants noted that intra-household allocation may be unequal. This inequality was said to be linked to gender and age, although not all participants agreed with this viewpoint. A few male participants said ASF consumption patterns are gender-equal. However, the general view from the study was that within households, men consume more ASFs than women and adolescents:

Well, I think both men and women eat meat a lot. As the woman is cooking, she'd be tasting and eating the meat. But some women recognize the fact that it is the man who bought the meat, so they will make sure they serve him well so that he'll continue buying meat for the household. So, for me, I think both men and women eat meat a lot **[“Antonio,” 48 years, Men's FGD#20, Volta Region].**

Due to menstrual flow in adolescents every month, they need more meat to give them protein and also to regain the lost blood. However, fathers take more meat in the family, but I think adolescents deserve more than any other person in the family **[“Yolanda,” 17 years, Adolescents' FGD#8, Northern Region].**

Many reasons were given to explain these gendered patterns of ASF consumption. Key among these reasons was a traditional perception that certain ASFs should be retained for men, most especially the thigh and liver of a chicken, and the head of all ruminants:

But there are some parts of ASFs that should always be served to men. If not, there will be trouble in the household. Like the chicken back, the thigh and the liver. Traditionally, all these parts should be served to the household head or elderly men **[“Tara,” 17 years, Adolescents' FGD#16, Volta Region]**.

As for the thigh of the chicken, the man is entitled to it. During the process of cooking, [women] continue to taste the meat and thereby end up consuming more. But at times, men consume more meat than the women. […] Men are also entitled to the liver **[“Travis,” 30 Years, Men's FGD#13, Northern Region]**.

To summarize, our analysis revealed that the major barriers shaping the consumption of ASFs include unequal intra-household allocation (e.g., by gender and age), affordability, and little knowledge about the nutritional values of these foods. It is possible that these reported barriers are influenced by level of education, poverty and socio-economic status. Given our FGD and KII data, however, we are unable to establish these associations, as we did not collect information on household wealth levels and educational background as determinants of ASF consumption. Nevertheless, there are numerous studies showing that better-off households are more likely than poorer households to purchase and consume ASFs (e.g., see Darapheak et al., 2013; Kaur et al., 2017). Available studies further show that households where members have attained at least a secondary education are more likely to consume ASFs, and also be aware of the nutritional benefits of these foods (Darapheak et al., 2013).

The most recent Ghanaian Consumer Price Index (CPI) indicated a marked upward trend of food prices since 2015, most notably for fish, meat, milk and eggs (Ghana Statistical Service, 2015a, Ghana Statistical Service (GSS) et al., 2015b). This higher CPI perhaps explains our respondents' concerns about the higher cost of ASFs, which leads to lower consumption of these foods. In 2015, for example, the cost of imported chicken was US$ 3 per kg, which increased to US$ 3.50 per kg in 2016 (Ashitey, 2017). Similarly, the cost of domestic chicken, a major preference for most Ghanaian households (Kwakwa, 2013; Woolverton and Frimpong, 2013), was US$ 12.80 per kg in 2015, which increased to ∼ US$ 15.00 per kg in 2016 (Ashitey, 2017). All these price trends add further credence to our study participants' perceptions that ASFs are not affordable for socioeconomically deprived families.

#### 3.2.3 Theme 3: Perceived livestock-based interventions to reduce anemia

To address the challenges described above, four major intervention pathways were suggested by participants: (1) social and behavior change communication; (2) proper livestock housing; (3) livestock-in-kind credit; and (4) hatchery management. All of these potential interventions were mentioned by study participants without any prompting.

##### 3.2.3.1 Perceived pathway 1: Social and behavior Change Communication

The most frequent response from interviews was that any livestock-based intervention should start with, or even solely focus upon, social and behavioral change communication (SBCC). Such intervention entails the use of communication to promote and facilitate behavior change and support the requisite social change for improving health outcomes (Aboud and Singla, 2012). One participant described such an intervention by saying:

For me, if I am going to tackle the problem of anemia, I will first tackle it from the individual community, especially by adding the social and behavioral change communication, SBCC. I would prioritize the social and behavioral change communication. Because you can give the person ten cows, if the person does not see the need for consumption, he or she may sell the cow. That may not target anemia **[“Jeff,” NGO Key Informant, Northern Region]**.

Among participants who recommended this intervention, more specific suggestions were made. Some emphasized that topics to be covered in SBCC should include basic information on the health and nutritional benefits of consuming ASFs. Similar to our earlier findings, poverty was tightly interwoven into the rationale behind this intervention pathway:

The poverty rate is high to the extent that the household rearing goats don't consume the goats. We have to educate people on the importance of consuming the animals they rear. […] Is like a cocoa farmer who has never tasted cocoa drink before. We should educate people on the importance of consuming some of the animals they rear **[“Jamie,” CBO Key Informant, Central Region]**.

Respondents more emphatically suggested how SBCC should be channeled, and who should be involved. Village chiefs, generally considered to be very influential, were said to be in a position to effectively convey behavior change messages. Other participants also suggested that SBCC must be conveyed in a more entertaining manner, using strategies that are familiar to local villagers. While storytelling and poems were mentioned, drama was the medium most often suggested:

[…] For SBCC, use drama to educate the people. In my experience, when you gather the people for a talk, they don't come but when you organize a drama for them, the people would come in their numbers **[“Sheena,” NGO Key Informant, Central Region]**.

##### 3.2.3.2 Perceived pathway 2: Improved livestock housing

Appropriate livestock housing was the second leading intervention pathway suggested by participants. When asked how livestock production or management could be changed to effectively reduce anemia, one respondent suggested:

The change should be about the way the animals are housed and fed. And we need assistance on these **[“Kristina,” 16 Years, Adolescents' FGD#9, Northern Region]**.

All participants described “appropriate” livestock housing using similar descriptors such as “spacious,” “well-ventilated,” “roofed,” and located away from household structures, but also secure enough to avoid thefts. In some cases, livestock housing was considered to be too close to human living quarters, especially kitchens and bedrooms, thus increasing infections, which were perceived to increase anemia. Perceived infection risks from livestock were associated with the need for more appropriate animal housing. Commonly mentioned infection concerns included skin rashes, malaria, and yaws. These perceived infection risks were more often mentioned by community participants, than the national, regional, and district stakeholders. Typical expression of these concerns included:

There are ticks that come when the chicks are hatched, these ticks can spread all over the house and when they enter your hair, you develop yaws **[“Marguerite,” 17 Years, Adolescent's FGD#2, Central Region]**.

As I have stated earlier, some of the disease can be transferred to man when you eat the meat of the sick animal. However, others are through contact. There are these tiny ticks hidden in the fur of the animals […]. So if the human is present, these ticks can move from the fowls to the human. Also, in this village, livestock housing is mostly constructed behind the kitchen, making it easier for the ticks to be transmitted to the human **[“Vincent,” 49 Years, Men's FGD#6, Central Region]**.

The following focus group excerpts shed further light on free-ranging livestock and perceived fecal contamination, particularly in a context where human open defecation is common:

**Focus Group Moderator***: Let's talk about the fowls now. What are some of the problems associated with chicken?*
**“Betty”***: As for fowl, it is not good at all.*
**Moderator***: Why?*
**“Betty”***: They have been eating feces.*
**Moderator***: The fowls eat human feces?*
**Respondents (all)***: Yes!*
**Moderator***: Where do they find the feces?*
**“Ruth”***: If you have a younger brother and he defecates somewhere, before you realize, the fowl will come and start eating it.*
**Moderator***: So where do your younger siblings defecate?*
**“Elsie”***: At the back of the house.*
**Moderator***: So what other problems are there?*
**“Mercedes”***: They [fowls] also eat our food. They like rice and maize especially.*
**“Maddie”***: When they get into your room, it is difficult sacking them.*
**Moderator***: Do you mean the kitchen?*
**All Respondents** (laughing)*: Yes!*
**[Adolescents' FGD#17, Volta Region]**

In general, participants revealed that although there is no systematic monitoring of human feces in areas where livestock wander, it is a well-known challenge. They expressed that providing appropriate livestock housing could help reduce infections linked to livestock contact with feces.

##### 3.2.3.3 Perceived pathway 3: Encourage livestock-in-kind credit

The third potential intervention mentioned was providing livestock-in-kind credit. Here, participants suggested that two or more animals (preferably a female and a male) should be distributed to beneficiary households with the understanding that, over time, a specified number of female offspring will be passed on to other beneficiaries as repayment. Until the repayment is made, the original livestock is not deemed as ‘owned’ by the first beneficiary. With this approach, participants perceived that ASFs would be more easily available and potentially be consumed. The distribution of chickens and small ruminants was frequently suggested for this intervention:

Normally, members in the villages are more prone to anemia. So, to reduce this problem through animal source foods, the government should give each household about 5 cockerels and 5 layers. The layers would lay eggs and we would educate the households to consume the eggs. Overtime, these households can give new chickens to additional households, so that more people can benefit **[“Jamie,” CBO Key Informant, Central Region].**

It is important to note that not all study participants, in both KIIs and FGDs, favored this intervention. Some expressed reservations about the sustainability of livestock-in-kind credit, often commenting that it might create a culture of dependency. Others also felt that this intervention strategy may not succeed in improving nutrition among low-income households, unless combined with nutrition education:

You can provide free livestock and ask households to pass them to others. But you have to tell them the rationale behind giving them the animals. You make them understand that they can sell some of the animals or the eggs, but they must also consume some. If you don't tell them, they would sell all the animals **[“Ruben,” Government Key Informant, Volta Region]**.

##### 3.2.3.4 Perceived pathway 4: Improved fowl hatchery management

The final intervention pathway suggested was to improve local hatcheries of day-old chicks, and the quality of feed and drugs given to poultry. This intervention was proposed in only two key informant interviews. As one participant suggested:

The government should help improve the local hatcheries of day-old chicks. Ghana Standard Board should also monitor that the feed given to farmers is of high standards. The drugs for the birds should also be monitored. Now you don't know if the drugs you are buying for your birds is of standard because these drugs are not checked **[“Maxwell,” Government Key Informant, Central Region]**.

Overall, no major differences were noted in the type of interventions proposed across the three regions. However, there were differences in responses by community participants versus those from the national, regional, and district stakeholders. On the one hand, the national, regional and district stakeholders more often favored SBCC. Community participants, on the other hand, emphasized livestock-in-kind credit, a suggestion that was remarkably consistent across the three regions.

## 4 Discussions

### 4.1 Relating study findings to existing literature

Our results suggest that producing animals for consumption is not a primary motivation for raising livestock or investing in enhancing livestock productivity in Ghana. Other competing demands are often prioritized, of which income generation is foremost. High poverty levels in Ghana may underlie this priority on income generation (Ghana Statistical Service, 2014). The proportion of Ghanaians living below the poverty line in 2014 was 18.8%, 50.4%, and 33.8% in the Central, Northern, and Volta Regions, respectively (Ghana Statistical Service, 2014; see also Table 1). Based on results from our study, raising livestock is often seen as a type of ‘bank account’ or savings. This finding is supported by research from other African contexts (e.g. Chingala et al., 2017; Sumberg and Lankoandé, 2013). For example, Sumberg and Lankoandé (2013) suggested that in rural areas, where banking and insurance services are limited, animals are kept as a ‘walking bank’ or ‘savings banks on the hoof’. In other studies where farmers have prioritized the various motivations for raising livestock (e.g. for income, milk, manure, draught power, meat, and dowry), income generation has always been ranked first, and meat consumption usually last (Chingala et al., 2017).

Aside from income generation, our results demonstrate that livestock and their products serve other social functions, which also reduce their importance for household consumption. Our results further indicate that even when livestock products are consumed, this tends to be highly regulated within the family. Specifically, young children and WRA, the demographic groups for which ASF consumption is most likely to be nutritionally beneficial, rarely consume these foods. An earlier study by Colecraft et al. (2006) reported similar findings in Ghana's Central, Brong Ahafo, and Upper East Regions, showing that barriers to ASF consumption included inequitable allocation among women and young children (see also a more recent review by Ruel et al., 2018).

Based on findings from our study, four specific interventions emerged as potential pathways for using livestock to reduce anemia in Ghana. Table 5 synthesizes the connections between the barriers identified and the interventions proposed. The red section of the table depicts identified/perceived barriers, and the green section illustrates proposed interventions. Each proposed intervention has strengths and limitations.

**Table 5:**
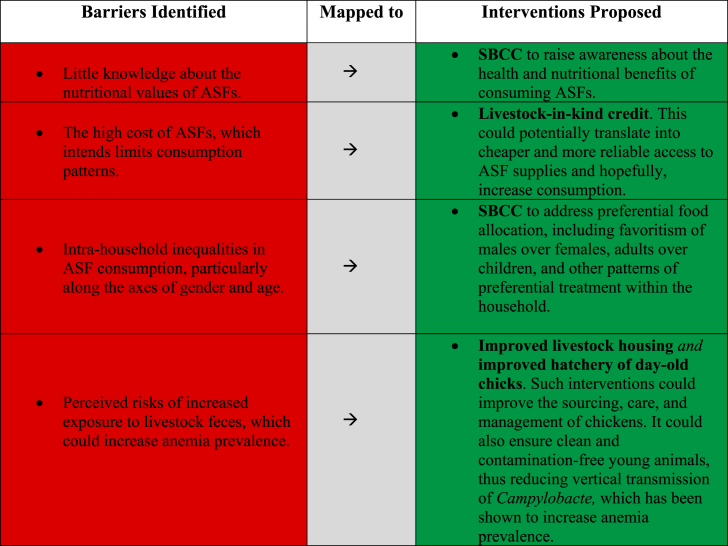
Connections between the barriers identified and interventions proposed.

Compared to other interventions, the first one, SBCC, requires relatively less technology and expense (Aboud and Singla, 2012). If appropriately contextualized, SBCC could effectively improve ASF consumption (Osei et al., 2016). However, studies have demonstrated that people are often not receptive to interventions aimed at changing dietary behavior (Barnes, 2015). Only a few examples of truly successful, sustainable, and cost-effective SBCC interventions promoting livestock and ASF consumption have been reported (Briscoe and Aboud, 2012). Often, structural barriers beyond the control of individuals are more crucial determinants of poor health than local attitudes and behaviors alone (Barnes, 2015). Furthermore, our study suggests an apparent tension between suggestions coming from community participants, and those of national, regional and district stakeholders. While the non-community stakeholders recommended SBCC to encourage ASF consumption, households in FGDs clearly indicated that livestock are needed for income, not for consumption. Thus, SBCC efforts encouraging own-consumption of livestock could produce unintended negative consequences for household incomes and associated health outcomes.

Improved housing and corralling of animals was the second intervention prioritized for reducing anemia through livestock production. Corralling domestic animals may reduce certain infections in people (e.g. *Campylobacter jejuni*), thereby decreasing diarrhea and anemia prevalence (Marquis et al., 1990). Contrasting evidence, however, indicates that corralling livestock may produce the opposite effect, and increase *Campylobacter*-related diarrhea in children (Oberhelman et al., 2006). Costs associated with improved livestock housing are also a concern. It is expensive to feed and maintain livestock kept partly or permanently in a corral. For example, a 2003 study in Peru showed that a simple poultry corral costs roughly US$ 50 (Harvey et al., 2003), yet households in Ghana's livestock-producing regions earn on average US$ 1.83 per day (Table 1). Such households may not easily invest one month's earnings in such a corral. Perhaps increasing poultry flock size could help address housing and feeding costs through more egg sales (Alemayehu et al., 2015). Another challenge is that animal housing may not be effective if implemented among only some households. Any benefits of corralling livestock from one household might be negated if animals from nearby households ran loose.

Livestock-in-kind credit, the third identified intervention, also has its own strengths and weaknesses. The NGO Heifer International has employed this intervention in many countries, with some documented benefits. If livestock-in-kind credit were to successfully reduce anemia, either through increased ASF consumption or increased income, the benefits accruing to the original recipients could spread more quickly to other vulnerable groups (Ayele and Peacock, 2003). This concept notwithstanding, results are mixed for actual nutritional impacts of livestock-in-kind credit programs. Only few studies have shown that these interventions actually increase ASF consumption and improve nutritional status (e.g., Rawlins et al., 2014; Ayele and Peacock, 2003). Moreover, investment costs are considerable in livestock-in-kind credit programs. For example, a recent study in Rwanda found that it costs ∼ US$ 3000 to give one cow to a household (Rawlins et al., 2014). Further, if farmers must provide their own initial housing and fodder, there is an additional cost of ∼US$ 300 (Kabumbuli and Phelan, 2003). In the Ghanaian rural contexts of our study, this cost could be prohibitive, and also extends beyond providing just the animals. To be sustainable, this intervention requires expenditures to improve animal feeding systems and training of community-based livestock health workers (Ayele and Peacock, 2003). Previous assessments of such an intervention showed that beneficiary households typically expect on-going support for housing, feeding, and animal disease management, thus putting a continuous strain on the initiating organization (Kabumbuli and Phelan, 2003). Given these limitations, and without broader investment in extension services, infrastructure, and institutions that support low-income farmers and livestock keepers, livestock-in-kind credit may not always be a feasible, cost-effective, or sustainable option.

Hatchery management, the final intervention identified, also represents an opportunity, but with constraints. Such an intervention could improve the sourcing, care, and management of day-old chicks, while ensuring clean and contamination-free young animals, thus reducing vertical transmission of *Campylobacter* and other pathogens (Kim and Kim, 2010). While hatchery management should improve chick health and survival, and reduce potential human contamination early in production, this intervention may not represent a comprehensive, long-term solution. It focuses solely on poultry early-life management, while neglecting post-hatchery husbandry practices. Also, it does not improve the broader range of livestock typically kept in Ghana, including goats, sheep, cattle, and pigs (FAOSTAT, 2018). From the results of our study, inefficient hatchery systems are not seen as the most important limitation to reducing anemia or improving ASF consumption in Ghana. Hatchery management would therefore likely need to be integrated with other interventions to effectively reduce anemia.

### 4.2 The specific contributions of this study

This study contributes to the growing literature and expanding debate on nutrition-sensitive agriculture, specifically how animal agricultural production and capture could be leveraged to improve nutrition (Berti and Cossio, 2017; Blackmore et al., 2018; Rawlins et al., 2014; Ruel et al., 2018). There are several research gaps in the evidence base of how livestock interventions could improve nutrition outcomes. For example, there is still little research on the characteristics of successful livestock-nutrition programs, or how successful programs could be delivered at scale (Ruel et al., 2018). As many reviews have shown over the past two decades (e.g. Blackmore et al., 2018; Leroy and Frongillo, 2007; Randolph et al., 2007), substantial challenges in operationalizing animal agricultural-nutrition linkages need to be overcome to better exploit potential opportunities. The findings from our investigation should enhance understanding of both the importance of animal agriculture for nutrition, and the conditionality of that importance on contextual factors. Specifically, we have shown that the social and economic context of livestock production and management must be thoughtfully considered in programs that seek to promote livestock production to increase ASF consumption. While the scientific literature suggests linkages among increased livestock production, greater income, and improved nutritional status (Murphy and Allen, 2003; Nicholson et al., 2003; Randolph et al., 2007), our study shows that there are complex, practical challenges to promoting these relationships. Any intervention must, therefore, consider the diverse pathways through which changes to animal production may influence anemia, including through changes in ASF consumption and risks of infection.

### 4.3 Study limitations

Our findings should be interpreted in context, taking into account the sample size and methodology used. Indeed, our overall research design is ‘rigorous’ in a qualitative sense, and our findings have been validated using member-checking (Miles et al., 2014). However, we caution against generalization of the results. Because of the purposive maximum variation sampling (Patton, 2014), selection bias is a potential limitation. The main purpose of this study was not to ascertain national trends in livestock production and management, or generalizable patterns of anemia across the country. Rather, our goal was to better understand the context-specific complexities of improving livestock production to reduce anemia through multiple pathways. We expect that these results will serve to generate hypotheses that can be more fully explored in larger-scale, probability-based sample surveys.

## 5 Conclusion and implications for livestock-related interventions

The findings reported here have several implications for interventions aiming to support livestock production and management, with an ultimate goal of reducing anemia among women. Overall, our results suggest the following recommendations and conclusions.

1. From our conceptual framework (Fig. 1), anemia reduction through improved nutrition is most likely to be affected by livestock production via two pathways involving increased ASF availability leading to increased direct consumption, and improved access to micronutrient-rich foods as a result of increased income. From the Ghanaian experience presented in this study, the first pathway is unlikely, unless efforts are made to purposefully encourage direct consumption of ASFs. The best potential lies in the second pathway; indeed, our results show that when income is generated from livestock, these funds are used to buy ASFs in smaller quantities, and also to pay for healthcare and other expenses that additionally should have direct and indirect impacts on anemia.
2. From the various interventions proposed by our study participants, the most feasible, sustainable, and relatively cost-effective appears to be an integration of proper livestock housing and SBCC. As indicated in our conceptual framework, nutritional pathways are not the only route through which livestock rearing may influence anemia. Exposure to microbial pathogens leading to subclinical and clinical infections is also an important pathway. Improving livestock housing could potentially help to reduce human infections caused by exposure to livestock and animal feces. These mechanisms are poorly understood in the Ghanaian context, yet have important implications for anemia risk. The integration and implementation of interventions to improve animal housing may directly address this pathway to anemia, but, as discussed above, there are several limitations. Livestock housing is very expensive, but costs could be minimized by using local materials. Furthermore, livestock housing interventions would need to address the hygiene of handling animals, which could be implemented using SBCC. However, not all livestock can be corralled, making SBCC all the more important for educating people on the potential health risks of allowing animals to free-range within household compounds and inside living quarters. If SBCC were to be implemented, it should be community-led as much as possible, with messages conveyed in a more entertaining way using channels relevant to local contexts. With careful and context-specific planning, it is possible for livestock production to make an important contribution to improving nutrition and mitigating anemia in Ghana and other developing country contexts.

## Footnotes

1 To protect participants' identity, all names are randomly generated pseudonyms.
